# Unlocking the power of probiotics, postbiotics: targeting apoptosis for the treatment and prevention of digestive diseases

**DOI:** 10.3389/fnut.2025.1570268

**Published:** 2025-03-31

**Authors:** Qiuyan Xie, Ji Liu, Ping Yu, Ting Qiu, Shanyu Jiang, Renqiang Yu

**Affiliations:** ^1^Department of Neonatology, Affiliated Women’s Hospital of Jiangnan University, Wuxi Maternity and Child Health Care Hospital, Wuxi, China; ^2^Department of Gastroenterology, The First Affiliated Hospital of Soochow University, Suzhou, China; ^3^Reproductive Medicine Centre, Affiliated Women’s Hospital of Jiangnan University, Wuxi, China; ^4^Department of Child Health Care, Affiliated Women’s Hospital of Jiangnan University, Wuxi, China

**Keywords:** probiotics, postbiotics, apoptosis, gut microbiota, digestive system diseases

## Abstract

Digestive diseases are becoming an increasingly serious health burden, creating an urgent need to develop more effective treatment strategies. Probiotics and postbiotics have been extensively studied for their potential to prevent and treat digestive diseases. Growing evidence suggests that programmed cell death, especially apoptosis, is a critical mechanism influencing the molecular and biological aspects of digestive diseases, contributing to disease progression. Understanding the mechanisms and signaling pathways by which probiotics and postbiotics regulate apoptosis could reveal new therapeutic targets for treating digestive diseases. This review focuses on the beneficial effects of probiotics and postbiotics in regulating apoptosis across a range of liver diseases, including non-alcoholic fatty liver disease, liver injury, cirrhosis, and liver cancer. It also explores their effects on gastrointestinal diseases, such as colorectal cancer, colitis, gastrointestinal injury, and infectious diarrhea. Furthermore, some probiotics help balance the gut microbiota, enhance intestinal barrier function, and regulate the immune system, all of which are closely associated with apoptosis. Moreover, emerging technologies, such as encapsulation methods, have been developed to stabilize probiotics, primarily based on experimental findings from rodent and human studies.

## 1 Introduction

**Figure 1:**
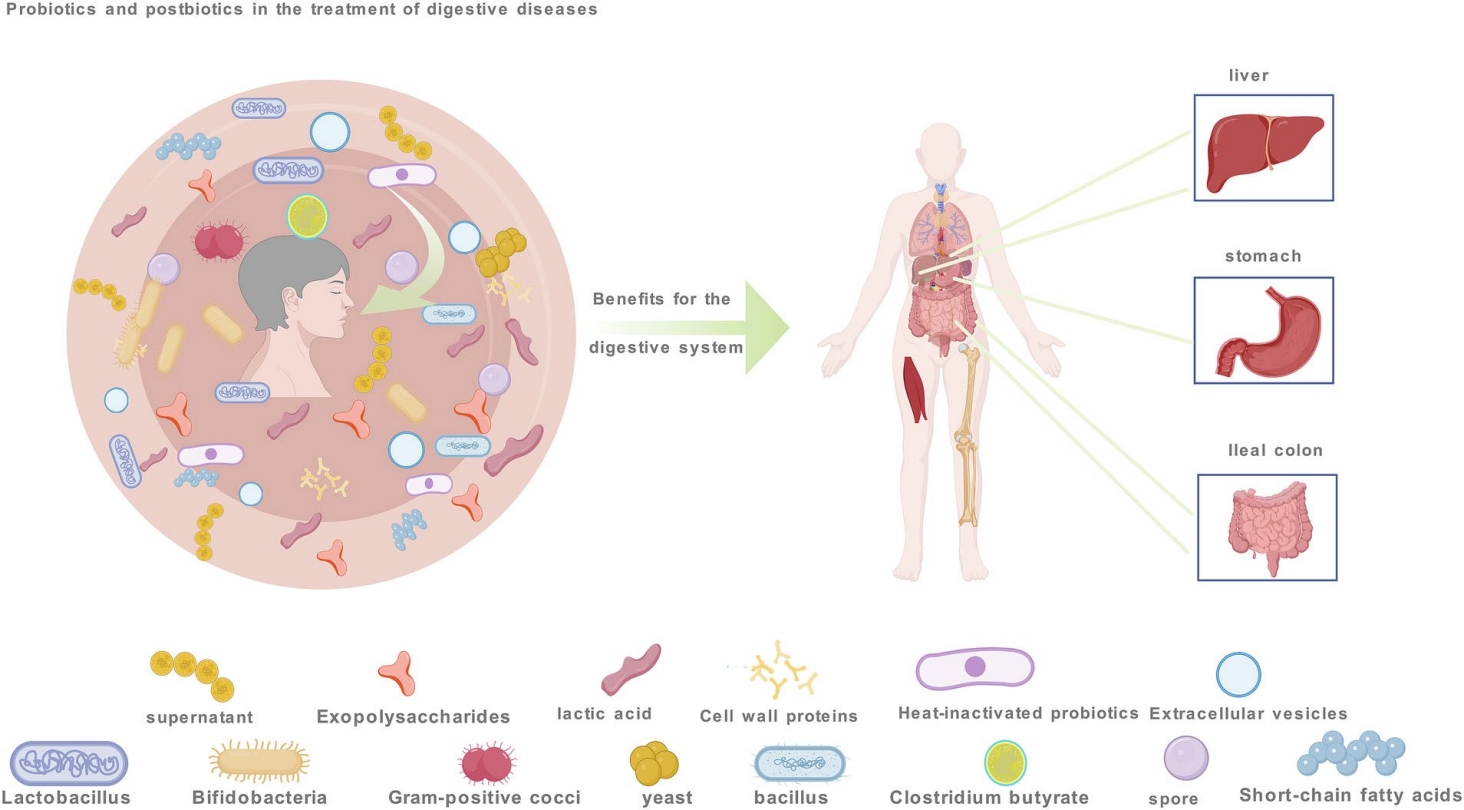
Probiotic and postbiotics aimed at improving gastrointestinal disorders by modulating apoptosis encompasses. These include probiotics like *Lactobacillus* and *Bifidobacterium*, *Gram-positive cocci, yeast*, *Bacillus*, *Clostridium butyricum*, postbiotics such as extracellular vesicles and lactic acid, spores and extracellular polysaccharides.

The age-standardized incidence of digestive diseases worldwide is 95,582 per 100,000, representing over one-third of the total prevalence of all diseases (1). Digestive disorders result in millions of medical visits and billions of dollars in economic costs annually in the United States (2). The prevalence of digestive diseases is rising globally, placing a significant burden on public health (3, 4). Despite significant advancements in medical devices and healthcare, many patients still experience poor quality of life and prognosis, highlighting the need for more treatment options. Recent studies have shown that both probiotics and postbiotics have gained significant attention for their potential to improve various digestive diseases by regulating host immune function, maintaining intestinal barrier integrity, and modulating the gut microbiota (5), as shown in Figure 1.

Apoptosis, or programmed cell death (6), is a crucial process that maintains tissue homeostasis by removing damaged or dysfunctional cells (7). However, its dysregulation is a critical factor in the development of digestive diseases (8–10). In inflammatory bowel disease, excessive apoptosis of intestinal epithelial cells disrupts the mucosal barrier, worsening inflammation and microbial translocation, while impaired apoptosis of immune cells prolongs chronic inflammation (11). In colorectal cancer, the promotion of apoptosis in cancer cells is driven by key genes such as P53, K-ras, and Bcl, which are associated with intrinsic apoptosis pathways. These mechanisms contribute to inhibiting tumor growth and reducing chemotherapy resistance (9, 12). In gastrointestinal infections, rotavirus enters mucosal epithelial cells via virulence factors such as sialic acid and histo-blood group antigens, triggering apoptosis. This process results in mucosal damage and delayed healing (13). These findings highlight the importance of targeting apoptotic pathways in developing therapeutic strategies for digestive diseases, with probiotics and postbiotics emerging as promising modulators.

Probiotics (it refers to active microorganisms that confer benefits to the host), either directly or through the secretion of metabolites or the regulation of host signaling pathways such as Bcl-2/Bax, caspase cascades, and key anticancer pathways, can inhibit excessive apoptosis or induce apoptosis in abnormal cells while protecting normal cells. These mechanisms contribute to alleviating pathological conditions, as seen with *Lactobacillus plantarum*, *Lactobacillus rhamnosus*, and others (9, 14, 15). Postbiotics (it is a collective term for the metabolic components of probiotics after processing), as functional metabolites of probiotics, can directly or indirectly target key nodes in the apoptosis process, including short-chain fatty acids, heat-killed probiotics, etc. (15, 16). How do probiotics and postbiotics regulate apoptosis in different types of gastrointestinal diseases?

## 2 Probiotics, postbiotics, and apoptosis

**Figure 2:**
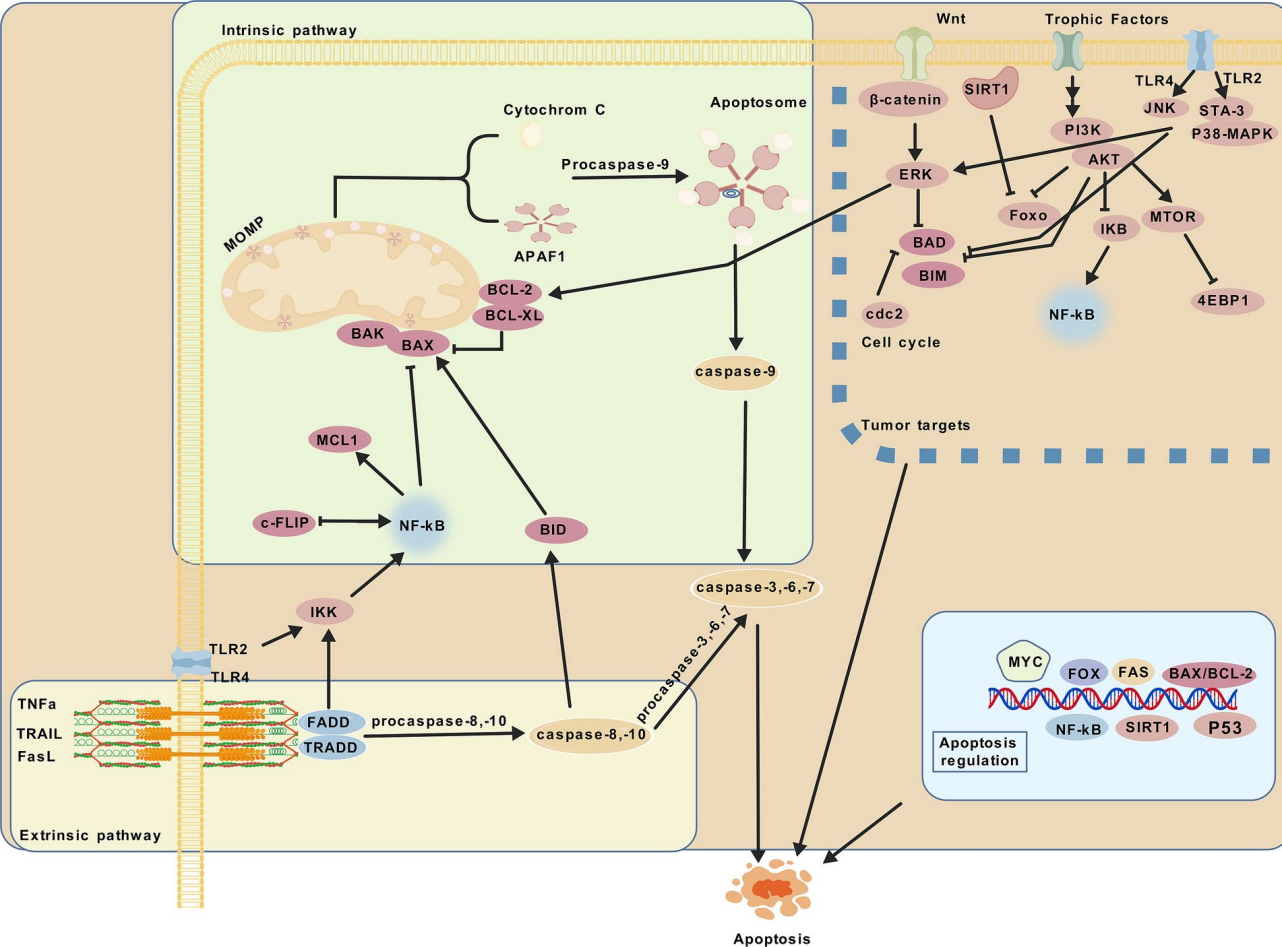
Modulation of apoptosis. Probiotic and postbiotics modulate apoptosis through extrinsic and intrinsic pathways, effectively influencing digestive diseases and targeting key pathways in tumors to promote cancer cell apoptosis.

Apoptosis is a crucial mechanism in the growth and development of multicellular organisms (7). The exogenous pathway, mediated by membrane receptors and regulated by Bcl-2 family proteins, and the endogenous pathway, mediated by mitochondria, are both closely linked to caspase regulation (17). When a cell detects internal abnormalities, it activates an intrinsic apoptosis program, initiating endogenous apoptosis. This process involves Bcl family proteins (pro-apoptotic members such as Bax, Bak, Bim, Bid, and PUMA, and anti-apoptotic members such as Bcl-2, Bcl-xl, Bcl-w, and MCL1) which alter the permeability of the mitochondrial outer membrane (18–22). This results in the release of cytochrome c, formation of the apoptosome with APAF1, and activation of caspase-9, which in turn activates apoptotic executor proteins caspase-3, -6, and -7, ultimately leading to apoptosis (23). The regulation of Bcl-2 protein transcription and phosphorylation in apoptosis involves CDK and p53 (24). The ERK1/2 and MAPK1 pathways promote cell survival partly by phosphorylating BIM, leading to its proteasomal degradation and inhibition of apoptosis (25). Probiotics are live, non-pathogenic microorganisms (26). This paper typically contains one or more microbial strains, with the main components including *Lactobacillus* spp., *Bifidobacterium* spp., Gram-positive cocci spp., yeast spp., *Bacillus* spp., and *Clostridium butyricum* spp. This paper highlights that most probiotics regulate apoptosis through intrinsic pathways, influencing the expression of Bcl family proteins, caspases, cytochrome c, mitochondria, and key anticancer pathways, such as the EGFR/PI3K/AKT signaling cascade, mTOR pathway, P38/JNK pathway, TLR4/JNK/NF-κB pathway, TLR4/MAPK pathway, Wnt/β-catenin pathway, and cAMP-dependent signaling pathway, as shown in Figure 2. When a cell receives an external death signal, exogenous apoptosis is triggered through a cascade of reactions. This process involves the binding of ligands (FASL, TNF-a, TRAIL) (27–31) to their corresponding receptors (FAS, TNFRs, TRAILRS) (23, 32–34) followed by a cascade of reactions that activate the cleavage of caspase-8 and -10. Subsequently, the cleavage of apoptosis executor proteins caspase-3, -6, -7 and BID is triggered, ultimately leading to apoptosis (35). BID serves as a link between the exogenous apoptosis pathway and the mitochondrial pathway (36), while the activation of promoter caspases is negatively regulated by c-FLIP (37). Probiotics observed in liver and colon cancer have been found to induce apoptosis through core apoptotic pathways similar to antitumor drugs, involving AKT, RAS, Raf, MEK, ERK, and mTOR kinase signaling pathways (38–42), and inhibiting growth factor receptors such as EGFR, Her2/Neu, other ERBB family members, c-Met, and NTRK (43–48), as depicted in Figure 2. Some probiotics regulate apoptosis through both endogenous and exogenous pathways, such as *L. plantarum C88*, including the toll-like receptor signaling pathway. Postbiotics refer to “inanimate microorganisms and/or their components that are beneficial to host health” (49), including heat-killed bacteria, extracts of extracellular polysaccharides (EPS) (50), extracellular vesicles (EVs) (51), cell wall protein components (52), spores (53), short-chain fatty acids (SCFAs) (9), lactic acid (54) and others, as depicted in Figure 1. They regulate multiple core signaling pathways associated with growth and development, control the production and function of apoptosis factors, reduce apoptosis in healthy cells, selectively promote apoptosis in cancer cells, and contribute to overall body health. Compared with active probiotics, postbiotics, with their microbial composition similar to pharmacological molecules, offer advantages in absorption, metabolism, and excretion (55), making them more reliable dietary supplements, such as heat-killed yeast (HKY) (56), and EVs of *L. rhamnosus PTCC1637* (51). The regulation of apoptosis may be intricately linked to certain bacterial components. Advancements in science and technology have facilitated the mass production of probiotics, such as the engineering of bacteria (e.g., *butyrate* synthesized by *E. coli Nissle 1917*). Furthermore, nanomaterials and microgels significantly enhance the precision of anti-cancer targeting of probiotics (57). As shown in Figure 2, probiotic and postbiotics regulate apoptosis to alleviate digestive diseases through both intrinsic and extrinsic pathways, promoting cancer cell apoptosis by targeting tumor-associated signaling pathways and key molecular targets.

## 3 Probiotics, postbiotics, and liver disease

### 3.1 Non-alcoholic fatty liver disease and liver injury

Non-alcoholic fatty liver disease (NAFLD) is a metabolic liver disorder unrelated to excessive alcohol consumption and is the most common chronic liver disease in Western countries (58). Recent studies have emphasized the important role of probiotics in treating and preventing liver diseases by modulating gut microbiota, strengthening the intestinal barrier, and interacting with the gut-liver axis (the gut-liver axis refers to the complex interplay between the gut and the liver, where gut microbiota-derived metabolites and immune-digestive interactions influence liver function, while liver-derived metabolites, in turn, regulate the gut microbiota and enhance intestinal barrier function) (59, 60). Apoptosis plays a crucial role (61). In a mouse model of NAFLD, *L. johnsonii BS15* was found to prevent the disease by regulating gut microbiota and modulating immunity. The anti-apoptotic effect was linked to reduced cytochrome c content and increased uncoupling protein-2 levels in mice (62). *Lc. paracasei HY7207* significantly alleviated symptoms and improved serum markers in mice with NAFLD. Additionally, the expression of Bcl-2 and Bax genes and their ratios were reduced, thereby decreasing apoptosis in human hepatocytes (8). As shown in Table 1, *Lactobacillus* spp. as dietary supplements, hold great potential for reducing endogenous apoptosis in the prevention and treatment of NAFLD. However, further studies are needed to determine whether they have similar effects in alcoholic fatty liver disease.

**Table 1:** Molecular mechanism of *Lactobacillus* spp. regulated apoptosis and improving liver injury.

Probiotic-based preparation	Category	Digestive diseases	The specific molecular mechanism of apoptosis	References
*Lc. paracasei HY7207*	Probiotic	Non-alcoholic fatty liver disease	Human: Regulated Bcl-2 and Bax-related genes and alter the Bax/Bcl-2 ratio	Kim et al. (8)
*L. johnsonii BS15*	Probiotic	Non-alcoholic fatty liver disease	Animal: Mitigated the reduction in cytochrome c levels and the increase in uncoupling protein-2 levels, preserving mitochondrial function	Xin et al. (62)
*L. paracasei GMNL-32*, *L. reuteri GMNL- 89 and L. reuteri GMNL-263*	Probiotics	Systemic lupus erythematosus liver injury	Animal: Inhibited the IKK/NF-κB signaling pathway downregulates the expression of caspase-3	Hsu et al. (64)
*L. reuteri ZJ617 culture supernatant*	Postbiotic	Acute liver injury	Animals: Inhibited the TLR4/MAPK signaling pathway downregulates the protein expression of caspase-3 and Bax	Cui et al. (65)
*Bacillus amyloliquefaciens SC06*	Probiotic	Acute liver injury	Animal: The downregulation of Bax, Caspase-3, Caspase-9, and p53 gene expression may be associated with the inhibition of the NLRP3 inflammasome	Wang et al. (67)
*Bacillus amyloliquefaciens B10*	Probiotic	Acute liver injury	Animals: Inhibited the expression of Bax, Bcl-2, and Caspase-3 gene and protein	Li et al. (68)
*L. plantarum C88*	Probiotic	Acute liver injury	Animals: Inhibiting the TLR2/NF-κB and TLR4/NF-κB signaling pathways can modulate the cell death receptor and mitochondrial pathways by downregulating the expression of Fas, FADD, TRADD, and Caspase-8, decreasing Bax and caspase-3 expression in liver cells while enhancing Bcl-2 expression	Huang et al. (14)

The liver, a vital metabolic organ, can be damaged by various factors, including infection, chemical toxicity, immune dysfunction, poor nutrition, circulatory issues, and genetic abnormalities (63). Supplementing with specific probiotics can alleviate the outcomes of autoimmune diseases by modulating immune responses and gut microbiota composition. In a liver injury model in lupus-prone mice, *L. paracasei GMNL-32*, *L. reuteri GMNL-89*, and *L. reuteri GMNL-263* were found to downregulate liver cell apoptosis and inflammation-related marker expression. This is closely linked to the inhibition of the MAPK and NF-kB signaling pathways, with the inhibition of the IKK/NF-κB pathway downregulating caspase-3 expression to reduce liver cell apoptosis, providing a basis for these preparations to serve as alternative drugs for liver diseases in systemic lupus erythematosus (64). Furthermore, the culture supernatant of *L. reuteri ZJ617* attenuates lipopolysaccharide (LPS)-induced acute liver injury by strengthening intestinal barrier integrity, modulating inflammatory responses, suppressing the hepatic TLR4/MAPK/NF-κB signaling pathway, and facilitating Beclin1-dependent autophagy. The anti-apoptotic effect, linked to downregulation of caspase-3 and Bax proteins, may result from suppression of the TLR4/MAPK signaling pathway (65). This suggests that different probiotics and postbiotics may alleviate liver injury through intersecting signaling pathways. However, further research is needed to determine whether the specific components involved are identical.

The NLRP3 inflammasome is a key component of the innate immune system (66)*. Bacillus amyloliquefaciens SC06* significantly inhibits the NLRP3 inflammasome and markedly reduces hepatocyte apoptosis (67). *Bacillus amyloliquefaciens B10* significantly reverses the gene and protein expression of Bax, Bcl-2, and Caspase-3 induced by aflatoxin B1, but lacks exploration of the underlying molecular mechanisms (68). *L. plantarum C88* has been shown to possess beneficial properties such as improving intestinal barrier function and inhibiting inflammation. It inhibited inflammation and excessive cell apoptosis mediated by the TLR2/NF-κB and TLR4/NF-κB signaling pathways, involving regulation of cell death receptors and mitochondrial pathways (14). These studies suggest a close correlation between using live *Lactobacillus* spp. or their supernatants to treat liver injury, enhance intestinal barrier function, and inhibit inflammation. Bacillus species present new insights into the mechanism of alleviating acute liver injury, particularly regarding apoptosis.

As shown in Table 1, live *Lactobacillus* spp. or their supernatants primarily affect the NF-κB and MAPK signaling pathways, reducing both endogenous and exogenous hepatocyte apoptosis. Further research is needed to explore the characteristics of other probiotics used for liver injury and their potential effects on apoptosis inhibition.

### 3.2 Liver cirrhosis and liver cancer

Globally, liver disease causes 40,000 deaths annually, representing 1% of global mortality (2,019 out of every 25 deaths worldwide). Hepatitis, alcoholic and non-alcoholic fatty liver disease, and chronic liver damage are closely associated with cirrhosis development. Further progression can lead to liver cancer, significantly burdening patients’ lives (69). The efficacy of chemotherapy should not overshadow its harmful effects on healthy cells. Probiotics and postbiotics have recently emerged as promising interventions for preventing and treating cirrhosis and liver cancer, and mitigating disease complications. In treating *Schistosoma mansoni*-induced cirrhosis, oral administration of probiotics such as *L. acidophilus ATCC 4356* and *L. delbrueckii subsp. bulgaricus DSM 20080*, or their fermented yogurt, significantly reduce worm burden, egg production, and granuloma size and number in mouse liver tissue. Significant improvements in oxidative stress and liver fibrosis were observed, along with downregulation of caspase-3 and Bax/Bcl-2 expression (70). However, oral administration of EPS derived from *L. acidophilus ATCC 4356* in hepatocellular carcinoma rats induced by diethylnitrosamine and gamma radiation exhibited potent immunomodulatory effects. This effect is attributed to the inhibition of the TLR2/STAT-3/P38-MAPK pathway related to inflammation; However, the specific mechanism of apoptosis regulation remains unclear (50). Initial findings suggest that probiotics modulate intestinal flora, reduce toxic metabolite accumulation, and influence the progression of metabolic disorders, including diabetes and insulin resistance. Recent studies demonstrate that adding an emulsion containing *L. rhamnosus NCIMB 8010* and *Pediococcus acidilactici NCIMB 8018* to liver cancer cells mitigates insulin resistance induced by free fatty acid accumulation, enhances cell viability, and regulates the Bax/Bcl-2 and caspase axes to improve mitochondrial dysfunction. The mechanism may involve inhibition of the fetal protein/TLR4/JNK/NF-κB pathway (71). *In vitro* experiments with the human liver cancer cell line HepG2 showed that EVs derived from *L. rhamnosus PTCC 1637* prevent liver cancer and significantly increase the apoptosis index (Bax/Bcl-2 ratio), inducing cancer cell death (51). Further research is required to elucidate the mechanisms underlying extracellular vesicle and probiotic-host interactions. These findings demonstrate that *Lactobacillus* and its components, such as extracellular vesicles and polysaccharides, primarily regulate endogenous apoptosis through the Bax/Bcl-2 and caspase axis. These components are promising alternative therapies for preventing and treating liver cancer, as shown in Table 2. As a critical step in the progression of liver cancer, the significance of cirrhosis should not be overlooked while exploring probiotic strains and molecular mechanisms involved in liver cancer.

**Table 2:** Active probiotics and postbiotic regulate apoptosis in the treatment of liver cirrhosis and liver cancer.

Probiotic-based preparation	Category	Probiotic type	Digestive diseases	The specific molecular mechanism of apoptosis	References
EVs derived from *L. rhamnosus PTCC1637*	Postbiotic	*Lactobacillus*	Liver cancer	Human: Increased the apoptotic index (Bax/Bcl-2 expression ratio)	Behzadi et al. (51)
*L. rhamnosus NCIMB8010* and *Pediococcus acidilactici NCIMB8018*	Probiotics	*Lactobacillus* Gram-positive coccus	Liver cancer	Human: Inhibited of fetoglobulin /TLR4/JNK/NF-κB axis, regulation of Bax/Bcl-2, caspase axis, improved mitochondrial function	Mularczyk et al. (71)
*L. acidophilus ATCC4356* and *L. delbrueckii subsp. bulgaricus DSM20080*	Probiotics	*Lactobacillus*	Schistosomiasis infectious cirrhosis	Animals: Regulated expression levels of caspase-3 and Bax/Bcl-2 in liver tissue	El-Khadragy et al. (70)
EPS derived from *L. acidophilus ATCC 4356*	Postbiotic	*Lactobacillus*	Liver cancer	Animals: Suppressed the TLR2/STAT-3/P38-MAPK path	Khedr et al. (50)

## 4 Probiotics, postbiotics, and colitis

The global prevalence of ulcerative colitis is rising, significantly affecting quality of life [not only affects the digestive system but also influences periodontitis (72)]. Effective treatment aims to induce and maintain remission (73). Current treatments include aminosalicylates, corticosteroids, antibiotics, adjunctive medications, and immunosuppressive agents; however, their efficacy is often suboptimal and tolerability limited. Exploring alternative treatments is essential (74). The ability of probiotics to regulate gut microflora, enhance the intestinal mucosal barrier, and improve immune function has positioned them as potential biological agents for treating colitis (75). In a Dextran Sulfate Sodium Salt (DSS)-induced mouse colitis model, the supernatant of *Lactobacillus GG-fermented* milk inhibited intestinal epithelial cell apoptosis, potentially by activating the PI3K/Akt pathway, which upregulates the anti-apoptotic protein Bad and inhibits FOXO transcription factors. This effect is linked to the unique p40 and p75 proteins in the probiotic-fermented milk supernatant (76). A pectin/zein hydrogel bead system improves protein delivery stability (77). Preliminary studies show that tumor necrosis factor-α (TNF-α) mediates inflammatory responses in inflammatory bowel disease. Its apoptotic role makes it a key target for destroying intestinal epithelial cells and a prime focus of pharmacotherapies. Oral administration of *L. BB12* and *L. plantarum LB-9* downregulates TNF-α expression and modulates caspase-8-mediated extrinsic apoptosis (78, 79). Secreted factors from *B. bifidum infantum 15697* reduce infections in a mouse model of necrotizing enterocolitis, prevent weight loss, and mitigate apoptosis caused by caspase-3 and caspase-7 activation, likely through NF-κB pathway inhibition (80). Combining probiotics with nanomaterials provides additional benefits, such as prolonged circulation and intestinal immunity regulation (81); *Bacillus amyloliquefaciens*-loaded nanoparticles enhance stability and improve endogenous apoptosis compared to free Bacillus. Specifically, They downregulate caspase-3 and cytochrome c expression while upregulating Bcl-2 and Bax, suggesting nanotechnology offers promising avenues for the food industry (82), suggesting that nanotechnology offers promising avenues for the food industry. These studies show that modulating the PI3K/Akt signaling pathway, regulating the TNF-α-mediated death receptor pathway, and inhibiting apoptosis through the Bcl-2 family and CytC-mediated mitochondrial pathway are key to the preventive and therapeutic effects of certain probiotics in colitis. Exploring novel probiotics and technologies that combine them with specific substances has further enhanced their potential benefits. As shown in Table 3, probiotics may serve as promising targeted therapeutic supplements. However, most studies still require further exploration of these novel formulations and more in-depth investigations into their molecular mechanisms, such as Limosilactobacillus reuteri FN041 (83).

**Table 3:** Probiotics, postbiotic regulate cell apoptosis and alleviate colitis.

Probiotic-based preparation	Probiotic type	Digestive diseases	The specific molecular mechanism of apoptosis	References
The supernatant of LGG-fermented milk	*Lactobacillus*	Colitis	Animal: Activated the EGFR/PI3K/Akt signaling pathway	Yoda et al. (76)
*Lactis Strain BB12*	*Lactobacillus*	Colitis	Animal: Down-regulated TNF-α expression suppressed caspase-8-mediated exogenous apoptosis	Chae et al. (78)
*L. plantarum LB-9*	*Lactobacillus*	Colitis	Animal: The down-regulation of TNF-α expression suppressed caspase-8-mediated exogenous apoptosis	Chae et al. (79)
The secreted factors of *B. bifidum infantum 15697*	*Bifidobacterium*	Necrotizing enterocolitis	Animal: Down-regulation of caspase-3 and caspase-7 activation	Weng et al. (80)
*Bacillus amyloliquefaciens*	*Bacillus*	Inflammatory bowel disease	Animal: The reduced levels of caspase-3 and cytochrome c, coupled with elevated Bcl-2 and Bax expression, led to increased endogenous cell apoptosis	Alkushi et al. (82)

## 5 Probiotics, postbiotics, and colorectal cancer

The prevalence of colon cancer has significantly increased in recent years due to poor dietary choices and unhealthy lifestyles, making it the second leading cause of cancer-related mortality worldwide (84). Recent studies suggest that probiotic preparations (it refers to probiotics and postbiotics) offer advantages over traditional drugs in certain cancer treatments and their side effects. When used as adjuvant therapy, probiotics can mitigate toxic side effects, optimize therapeutic outcomes, and enhance gut microflora, intestinal barrier function, and immune response (85). Inducing apoptosis in cancer cells is a key goal in advancing cancer treatments (86). A study using *S. cerevisiae* in a mouse model of colorectal tumors to inhibit cancer progression suggests that the downregulation of the Akt/NF-κB and Akt/mTOR signaling pathways may be linked to apoptosis in cancer cells. It also suggests a potential increase in beneficial gut microbiota and immune function regulation (87). Identifying specific *S. cerevisiae* strains with anti-colon cancer properties is crucial, as different strains have distinct therapeutic benefits (88). *The L. paracasei K5* strain adheres to human intestinal cancer cells, potentially inducing apoptosis by regulating Bcl-2 family proteins (89). Administration of *L. casei ATCC393* suppresses tumor growth and enhances apoptosis in mouse (CT26) and human (HT29) colon cancer cells by upregulating TRAIL expression and downregulating Survivin (90). The link between cell cycle regulation and apoptosis induction is well established (91). *L. paracasei subsp. paracasei X12* inhibits the mTOR/4EBP1 signaling pathway, induces G1 phase arrest in HT-29 cells, suppresses cyclin E1 expression, upregulates p27, and modulates apoptosis (92). The cell wall protein component of *L. paracasei ATCC25598* has been shown to mitigate apoptosis in the human intestinal Caco-2 cell line (52). This highlights the specificity of strains in regulating apoptosis mechanisms. Long-term retention of probiotics in the gut may improve disease prognosis, including cancer. *L. plantaris*, *L. rhamnosus*, *L. breve*, and *L. luciferi* extracted from human feces have anti-cancer effects by activating the Wnt/β-catenin pathway, antimicrobial peptides, and metabolites. These metabolites disrupt mitochondrial membrane integrity and trigger late apoptosis in tumors following colonization (93). Promoting the long-term presence of probiotics in the gut may improve disease outcomes, such as cancer prognosis. Similarly, a comparable study found that *L. sali*var*ius CGMCC3606* inhibits both early and late tumor formation in mice. Notably, its metabolites effectively suppress the AKT signaling pathway by inhibiting phosphorylation of AKT, cyclin D1, and COX-2, leading to apoptosis (94). But further studies are needed to understand how *Saccharomyces burra* metabolites promote cell apoptosis (95). These findings suggest that a single active *Lactobacillus* and its metabolites can induce apoptosis in cancer cells through various signaling pathways, offering promising prospects for clinical applications. Cancer development and progression are closely linked to chronic inflammation, a major contributing factor (96). *L. helveticus NS8* significantly reduces tumor number and proliferation in colitis-associated colorectal cancer mice, while promoting the increase of beneficial microbiota. Upregulating caspase-3 to promote apoptosis may involve inhibiting NF-κB activation and modulating inflammatory factors (97). Recently, there has been increasing focus on the immune function and molecular mechanism of non-viable probiotics rendered inactive by physical or chemical methods, such as heat and ultraviolet radiation, in relation to diseases (49). Notably, it possesses greater stability compared to live probiotics, making it highly advantageous as a food additive (98). Compared to 5-FU, heat-inactivated *S. cerevisiae PTCC5052* downregulates p-Akt1, Bcl-XL, Rel A, pro-caspase-3, and − 9, and enhances Bax and caspase-3 expression to induce cell apoptosis. The former has a more pronounced effect on Bax regulation through the Akt/NF-κB signaling pathway (56). Heat-induced apoptosis of human colorectal adenocarcinoma HT-29 cells depends on factors like time, dose, and specific strains, such as *L. brevis IBRC_M1078* and *L. paracasei IBRC_M1079*. These probiotic strains promote apoptosis by enhancing the expression of pro-apoptotic genes like Bax, caspase-3, and caspase-8, while suppressing the anti-apoptotic gene Bcl-2 (99). The purification of probiotics and the use of their cell-free supernatant should not be overlooked (16). The cell wall protein component of *L. paracasei ATCC25598*, for example, induces apoptosis in intestinal Caco-2 cells and may serve as an anticarcinogenic agent (52). The effect of *Lactobacillus* cell-free supernatant (LCFS) on apoptosis induction in human colon cancer cells was observed in a 3D colorectal cancer model, where it inhibited NF-κB activation and downregulated PARP1 and Bcl-XL expression (100). This offers a novel approach to investigating the anticancer properties of probiotics across various cancer types. With many chemotherapy drugs used in tumor treatment, drug resistance has emerged as a major factor contributing to the decline in drug efficacy. Recently, the use of probiotic components and metabolites has been found to effectively mitigate this issue. For example, *L. plantarum CCARM0067*, which produces γ-aminobutyric acid (GABA), shows anti-cancer effects on 5-fluorouracil-resistant human colorectal adenocarcinoma cells by inducing apoptosis through cIAP2 regulation and inhibition of the cAMP-dependent signaling pathway (15). The cell-free culture supernatant of this strain boosts the cancer-inhibiting effects of SMCT1/butyric acid in colorectal cancer cells, making it a potential chemotherapy enhancer for HCT116 cells resistant to 5-Fluorouracil and butyric acid. It is also closely linked to the activity pattern of caspase-3 (101). These findings offer a novel approach to chemoprevention and treatment of colorectal cancer-related diseases.

Various probiotic mixtures regulate apoptosis to improve disease outcomes. *L. plantarum AdF10* and *L. rhamnosus ATCC53103* enhance oxidative stress resistance and normalize p53-mediated apoptosis gene expression, potentially safeguarding against stress-induced excessive apoptosis. This may improve cellular health and reduce diseases associated with uncontrolled apoptosis (102). Celecoxib suppresses the AKT pathway, leading to decreased CD133 expression in colon cancer (103). The combination of *L. acidophilus NCDC15* and *L. rhamnosus GG MTCC1408*, along with celecoxib, was observed to decrease tumor heterogeneity and enhance immune function and gut health. It upregulates P53 expression, downregulates K-ras proto-oncogene expression, and modulates Bax and Bcl-2 levels, potentially inhibiting tumor growth and promoting overall health (12). This suggests that the molecular mechanism of apoptosis underlying the effects of chemotherapy may be altered when combined with probiotics. This combined approach may help alleviate the severity and burden of diseases in highly susceptible individuals. However, clinical validation is required.

Recent research shows that SCFAs, such as acetate, propionate, and butyrate, act as metabolites for gut bacteria to metabolize dietary fiber. These metabolites play crucial roles in inflammation, immunity, lipid metabolism, apoptosis mechanisms, and the regulation of key targets related to disease prevention and outcomes (104). To mitigate rapid clearance and enhance bioavailability, novel short-chain fatty acid analogs (105) and probiotics combined with nanomaterials (106) have been developed. The application of engineered bacteria is highly promising. Engineered *E. coli Nissle 1917* with synthetic butyrate reduced tumor volume by 70% in mice and induced apoptosis in human colorectal cancer cells through the mitochondrial pathway, independent of P53. This represents a novel approach to targeted bacterial cancer therapy (107). The latest study on microcapsules enables probiotics to exert targeted tumor therapy (108). Microencapsulated *L. plantarum LAB12* significantly reduces tumor volume and weight, inhibiting angiogenesis. The anti-apoptotic effect is partially linked to upregulation of p53 and caspase-3 expression (109). In recent years, *L. reuteri* has shown great promise in the treatment of various digestive diseases (110). The use of *L. reuteri* delivered *via* microgel technology in colorectal cancer enhances beneficial bacterial flora, increases butyrate production, and modulates the caspase and Bcl pathways to induce apoptosis in human colorectal cancer cells (57). This technology also enhances the gastrointestinal tolerance of *L. reuteri*. *Lactobacillus paracasei strain CMU-Pb-L5* and *L. reuteri* promote cancer cell apoptosis through similar mechanisms. Future research should focus on a more detailed investigation of the specific bacterial components involved in tumor growth inhibition (111). Acetyl-ethyl extract from *L. plantarum* ATCC14917 and *L. rhamnosus* ATCC7469 exhibits targeted anti-colon cancer cell activity by inducing the intrinsic apoptosis pathway, downregulating the expression of anti-apoptotic genes Bcl-2 and Bcl-XL, and upregulating the expression of pro-apoptotic genes Bak, Bad, and Bax (9). It is a potential candidate for aiding in the fight against cancer from a nutritional perspective. The studies indicate similarities in the targeted anti-cancer mechanisms of short-chain fatty acids derived from *Lactobacillus*, particularly in their ability to predominantly activate the intrinsic pathway to induce apoptosis in cancer cells. Compared with healthy individuals, intestinal conjugated linoleic acid (CLA) is significantly reduced in CRC patients. Besides fecal microbiota transplantation (FMT) (112), exogenous supplementation with CLA-producing *Bifidobacterium breve CCFM683* and *Bifidobacterium pseudocatenulatum MY40C* significantly inhibits tumor progression, which is closely associated with CLA and the bbi gene responsible for its production. *CCFM683* enhances intestinal barrier function by suppressing the NF-κB signaling pathway and promotes tumor cell apoptosis through the CLA-PPAR-γ axis (113), a mechanism consistent with the tumor-suppressive effects previously observed in *L. plantarum CCFM8661* (114).

Recent studies have found that new probiotics are effective in colorectal cancer. The novel probiotic strains *Streptococcus sali*var*ius CP163* and *S. salivarius CP208*, originating from human colostrum, demonstrate multifaceted anti-cancer properties. These include directly adhering to cancer cells, secreting short-chain fatty acids, inducing cancer cell DNA fragmentation and morphological alterations, modulating caspase-2 activity, and triggering apoptosis. This study unveils an innovative biological strategy for using functional foods in colon cancer prevention (115). Additionally, *in vitro* experiments have shown that *Ligilactobacillus salivarius LZZAY01* promotes cancer cell apoptosis (116). In summary, as shown in Table 4, probiotics or postbiotic play a crucial role in exerting anti-colorectal cancer effects through various signaling pathways related to tumorigenesis or by directly promoting the expression of proteins involved in cell apoptosis. Compared to conventional chemotherapeutic agents, microbial preparations have minimal or absent toxic side effects, providing them with a significant advantage in biological applications. The development of novel technologies, such as microencapsulation and nanomaterials, has further enhanced the stability and tumor specificity of these microbial preparations, which are used as nutritional dietary supplements for colorectal cancer prevention and treatment. Therefore, due to their excellent gastrointestinal tolerance and biological safety profiles, these microbial preparations are expected to have broader clinical applications.

**Table 4:** Probiotic and postbiotic promote apoptosis to alleviate colon cancer.

Probiotic-based preparation	Category	Probiotic type	The specific molecular mechanism of apoptosis	References
*L. paracasei K5*	Probiotic	*Lactobacillus*	Human: Potentially inducing apoptosis by regulating Bcl-2 family proteins	Chondrou et al. (89)
*L. casei ATCC393*	Probiotic	*Lactobacillus*	Human, animal: Induced up-regulation of TRAIL and down-regulation of survivin	Nychas et al. (90)
*L. paracasei subp. Paracasei X12*	Probiotic	*Lactobacillus*	Human: The blockade of the mTOR/4EBP1 pathway causes HT-29 cancer cells to pause in the G1 phase, reduce cyclin E1 expression, elevate p27 levels, and initiate apoptosis	Huang et al. (92)
The cell wall protein component of *L. paracasei ATCC25598*	Postbiotic	*Lactobacillus*	Human: The specific mechanism underlying the augmentation of cancer cell apoptosis remains unclear	Nozari et al. (52)
Heat-inactivated *S. cerevisiae PTCC5052*	Postbiotic	*Saccharomyces*	Human: Down-regulates p-Akt1, Bcl-XL, Rel A, and pro-caspase-3, -9, and enhance Bax and caspase-3 expression	Shamekhi et al. (56)
heat-killed *L. brevis IBRC_M1078* and *L. paracasei IBRC_M1079*	Postbiotic	*Lactobacillus*	Human: Pro-apoptotic genes, including Bax, caspase-3, and caspase-8, are upregulated, whereas the anti-apoptotic gene Bcl-2 is downregulated	Karimi et al. (99)
*L. genera (L. plantarum, L. rhamnosus, L. brevis and L. Lui)* and their metabolites	Probiotics Postbiotic	*Lactobacillus*	Human, animal: Activation the Wnt/β-catenin pathway, AMPs and metabolites, which are continuously generated in tumors following colonization, disrupt the mitochondrial membrane integrity and trigger late apoptosis	Ghanavati et al. (93)
*S. burra* metabolites	Postbiotic	*Saccharomyces*	Human: Promote apoptosis of cancer cells	Pakbin et al. (95)
*LCFS*	Postbiotic	*Lactobacillus*	Human: The activation of NF-κB was suppressed, while the expression of PARP1 and Bcl-XL was downregulated	Yoo et al. (100)
Gaba-producing *L. plantarum CCARM0067*	Postbiotic	*Lactobacillus*	Human: Regulation of cIAP2 expression and inhibition of the cAMP-dependent signaling pathway	An et al. (15)
Cell-free culture supernatant of *L. plantarum CCARM0067*	Postbiotic	*Lactobacillus*	Human: Regulated caspase-3 activity	Kim et al. (101)
*Butyrate synthesized by engineered colibacillus Nissle 1917*	Postbiotic	*E. coli*	Human: The induction of mitochondrial apoptosis pathway is P53-independent, resulting in up-regulation of cytochrome C, Bax, and PARP-1 protein expression. Additionally, activation of caspase-3 and caspase-9 occurs	Chiang et al. (107)
*L. reuteri*	Probiotic	*Lactobacillus*	Human: The expression of Bcl-2 was downregulated, while the expression of Caspase-3 and Bax was upregulated	Li et al. (57)
*(L. p. CMU-Pb-L5)*	Probiotic	*Lactobacillus*	Animal: The protein expression of Bcl-2 was downregulated, while the expression of Caspase-3 and Bax was upregulated	Chang et al. (111)
CLA-producing *Bifidobacterium breve CCFM683* and *Bifidobacterium pseudocatenulatum MY40C*, *L. plantarum CCFM8661*	Probiotics Postbiotic	*Bifidobacterium* *Lactobacillus*	Animal: Increased the concentration of the pro-apoptotic protein Bax and reduced the anti-apoptotic protein Bcl-2 through the CLA-PPAR-γ axis	Chen et al. (113, 114)
The supernatant from fermenting Musa paradisiaca with *L. casei NCDC17 and B. bifidum NCDC255* is rich in SCFA	Postbiotic	*Lactobacillus* *Bifidobacterium*	Human: Lowering mitochondrial membrane potential and ATP synthesis induces mitochondrial pathway-mediated cell apoptosis: release of cytochrome C, activation of BAX, increased expression of caspase-3 and PARP, without affecting BCL-2 expression	Nie et al. (145)
Acetyl-ethyl extract from *L. plantarum ATCC14,917* and *L. rhamnosus ATCC7469*	Postbiotic	*Lactobacillus*	Human: Downregulating the expression of anti-apoptotic genes Bcl-2 and Bcl-xl, and upregulating the expression of pro-apoptotic genes Bak, Bad, and Bax	Amin et al. (9)
*S. salivarius CP163* and *S. salivarius CP208*,	Probiotics	Gram-positive coccus	Human: Inducing cancer cell DNA fragmentation and morphological alterations, modulating caspase-2 activity, and triggering apoptosis	Srikham et al. (115)
*Ligilactobacillus salivarius LZZAY01*	Probiotic	*Lactobacillus*	Human: The protein expression of Bcl-2 was downregulated, while the expression of Bax was upregulated	Wenhong Yang et al. (116)
*S. cerevisiae*	Probiotic	*Saccharomyces*	Animal: Down-regulation of caspase-3 and caspase-7 may be linked to the down-regulation of the Akt/NF-κB and Akt/mTOR signaling pathways	Li et al. (87)
*L. helveticus NS8*	Probiotic	*Lactobacillus*	Animal: The upregulation of caspase-3 expression may be associated with the inhibition of NF-κB pathway activation	Rong et al. (97)
*L. salivarius CGMCC3606* and its metabolites	Probiotic Postbiotic	*Lactobacillus*	Animal: The AKT signaling pathway was inhibited, resulting in down-regulation of AKT phosphorylation and decreased expression of cyclin D1 and COX-2	Dong et al. (94)
*L. plantarum AdF10 and L. rhamnosus ATCC53103*	Probiotics	*Lactobacillus*	Animal: Restoring normal levels of p53, p21, and apoptotic genes (Bax, Bcl-2, caspase-3, and caspase-9) can suppress apoptosis via the p53 pathway	Walia et al. (102)
(*L. acidophilus NCDC15* and *L. rhamnosus MTCC1408*) along with cecxib	Probiotics	*Lactobacillus*	Animal: Upregulation P53 expression, downregulation K-ras proto-oncogene expression, and modulation Bax and Bcl-2 levels	Sharaf et al. (12)
*L. plantarum LAB12*	Probiotic	*Lactobacillus*	Animal: Upregulation of p53 and caspase-3 expression	Peng et al. (109)

## 6 Probiotics, postbiotic, and gastrointestinal damage

Encountering harmful substances and microorganisms is inevitable in daily life. When the immune system weakens, direct or indirect contact with the gastrointestinal damage system can the intestinal barrier, leading to microbiota imbalance, intestinal damage, and diarrhea. Historically, gastrointestinal injuries were mainly treated with pharmaceuticals and surgery; recent studies have shown that probiotics can offer protection. Maintaining the intestinal barrier function is crucial for gastrointestinal health, as shown in various studies (117). An *in vitro* study showed that *Clostridium tyrobutyricum* protects porcine epithelial cells from lipopolysaccharide-induced injury by preserving intestinal barrier function and inhibiting the P38/JNK signaling pathway. Downstream genes, including AP-1, ELK-1, ATF-2, and p53, are downregulated, while Stat3 activates anti-apoptotic Bcl-2 and downregulates pro-apoptotic Bax and caspase-3/-8, reducing intestinal cell apoptosis (118). Ochratoxin A, a significant toxin for humans and animals, has been detected in *in vitro* models of Ochratoxin A-induced cell injury. *Bacillus subtilis CW14* upregulated DNA repair genes and downregulated death receptor pathway genes to reduce apoptosis. These effects may be mediated by activation of toll-like receptor signaling pathways (119). The specific mechanism requires further investigation. Cadmium, a heavy metal, can damage various bodily systems through long-term environmental exposure, with the gastrointestinal tract being the primary target organ (120, 121); Administration of multi-strain probiotics (*L. rhamnosus IRBC_M10783*, *L. helveticus TG_34*, *Lactobacillus casei IRBC_M10782*) significantly reduces intestinal tissue damage in mice compared to untreated controls. Probiotics modulate immune function by upregulating p53, Bax, and caspase-3, while downregulating Bcl-2 to reduce apoptosis (122). Similarly, *Lactobacillus rhamnosus*, *Lactobacillus fermentum*, and *Lactobacillus brevis* inhibit indomethacin-induced mucosal cell apoptosis. Future research should focus on a more detailed investigation of the molecular mechanisms underlying the probiotic effects (123).

Research has shown that lactic acid, a *Lactobacillus* metabolite, mitigates ethanol-induced gastric mucosal damage by reducing local inflammation. It protects the stomach by inducing apoptosis through downregulation of IL-1β, TNF-α, and IL-6, as well as Bax and caspase-3, and by upregulating genes maintaining gastric mucosal integrity (10). However, the underlying mechanism remains unclear, and further research is needed to explore additional applications of probiotics in this field (54). Radiotherapy is a common and effective treatment for gastrointestinal tumors, but it inevitably damages adjacent healthy tissue (124). Restoring gut microbiota can partially alleviate this damage through fecal transplantation and probiotic therapy. The key is finding ways to mitigate the impact of stomach acid and ionizing radiation on the microbiota (125, 126). A recent study on probiotic spore layers (spore ghosts) showed that oral administration of three clinically approved probiotics (*Bacillus coagulans*, *Bacillus subtilis*, and *Bacillus licheniformis*) significantly enhanced the population of beneficial intestinal flora in mice. This effect was attributed to their exceptional stomach acid tolerance and biocompatibility. They also improved intestinal barrier function and reduced radiation-induced apoptosis in intestinal epithelial cells (53). These studies collectively indicate that apoptosis is a key mechanism through which various probiotics and their metabolites to prevent and treat gastrointestinal injury. Certain forms, such as spores, enhance gastrointestinal tolerance and play a crucial role in maintaining the integrity of the gastrointestinal barrier. Consequently, they hold promising potential as candidates for addressing gastrointestinal injury, as illustrated in Table 5. However, further research is needed to explore the use of additional probiotics, particularly through clinical trials.

**Table 5:** Probiotics and postbiotic regulate endogenous and exogenous cell apoptosis to alleviate gastrointestinal injury.

Probiotic-based preparation	Category	Probiotic type	The specific molecular mechanism of apoptosis	References
*Bacillus subtilis CW14*	Probiotic	*Bacillus*	Human: By activating the Toll-like receptor signaling pathway, upregulating DNA repair genes, and downregulating genes related to the death receptor pathway	Peng et al. (119)
*Clostridium tyrobutyricum*	Probiotic	*Clostridium butyricum*	Animal: Inhibition the P38/JNK signaling pathway, downregulation downstream genes (including AP-1, ELK-1, ATF-2, and p53), activation Stat3 expression, thereby regulating the Bcl and caspase families	Xiao et al. (118)
*L. rhamnosus IRBC_M10783, L. helveticus TG_34, L. casei IRBC_M10782*	Probiotics	*Lactobacillus*	Animal: The endogenous pathway of upregulating the expression of p53, Bax, and caspase-3 genes while downregulating Bcl-2 gene expression contributes to the reduction of cellular apoptosis	Dashtbanei et al. (122)
*Lactobacillus* metabolites: lactic acid	Postbiotic	*Lactobacillus*	Animal: By alleviating cell apoptosis induced by inflammatory cytokines and downregulating the expressions of Bax and caspase-3	Huang et al. (54)
*L. rhamnosus, L. fermentum*, and *L. brevis*	Probiotics	*Lactobacillus*	Animal: The up-regulation of Bcl-2 expression and the down-regulation of Bax expression	Gelen et al. (123)
Spores of *Bacillus coagulans, Bacillus subtilis and Bacillus licheniformis*	Probiotics	*Bacillus*	Animal: The apoptosis rate can be decreased	Zheng et al. (53)

## 7 Probiotics, postbiotic, and infectious diarrhea

In the United States, acute diarrheal diseases cause approximately 179 million outpatient visits annually (127). Probiotics and postbiotics, along with rehydration, medication, and improved hygiene, offer a promising approach for controlling early-stage gastrointestinal infections, providing a significant solution to this global health issue (128, 129). They can significantly modulate gut microbiota, enhance intestinal barrier function, and regulate immune responses, thereby exerting probiotic properties to alleviate diarrhea. Rotavirus is widely recognized as the leading cause of severe gastrointestinal infections in infants and children (130). In an experimental study with viral intervention in weaning piglets, *LGG* was found to significantly alleviate diarrhea caused by viral infection, upregulate the Bcl-2 gene, and downregulate the Bax gene, reducing apoptosis of jejunal mucosal cells (131). A recent *in vitro* study using a human model showed that both *LGG* and its conditioned medium (*mLGG*) reduced elevated caspase-3 activity, potentially through inhibition of the NF-κB signaling pathway. Their benefits differ: live *LGG* primarily suppresses enterotoxic and cytotoxic effects, while *mLGG* exerts postbiotic effects mainly by inhibiting chloride ion secretion pathways (129). The findings suggest that the mechanism through which *LGG* inhibits apoptosis and alleviates virus-induced diarrhea may be species-specific. Additionally, the addition of *Bacillus clausii* mixed strains (*O/C*, *T, SIN*, *N/R*) and their supernatant reduced intestinal cell apoptosis rates. This mechanism may involve activation of the cellular TLR3 pathway and suppression of NF-κB1, TRAF6, and MyD88. These findings encourage further exploration of the effects of *Bacillus clausii* on gastrointestinal infections caused by other pathogens in future studies (132). The 3D8 single-chain variable region protein shows potential antiviral activity by penetrating cells and degrading nucleic acids. Oral administration of *L. paracasei ATCC334*, which produces recombinant 3D8 single-chain variable region protein, reduces norovirus coat protein VP1 expression and increases the expression of the anti-apoptotic protein survivin in mice (133). *Salmonella typhimurium* is a prevalent foodborne pathogen, while *Bacillus subtilis Gbi-30*, *Bacillus indium ATCC6633*, and *Bacillus coagulans IBRC-M10981* inhibit its growth in spore and heat-inactivated forms without toxic effects on intestinal cells. These strains are recommended for the prevention and treatment of disease (134). These studies show that probiotics and postbiotics can reduce gastrointestinal cell apoptosis induced by viral or bacterial infections, as shown in Table 6. This effect is likely due to their anti-inflammatory properties, maintenance of intestinal barrier function, and direct pathogen-targeting mechanisms. However, the precise molecular mechanism through which probiotics reduce gastrointestinal cell apoptosis following viral infection remains unclear. Future research should explore the effects and mechanisms of different probiotic strains on various pathogens, such as *Helicobacter pylori*-induced gastrointestinal infections, and their impact on intestinal cell apoptosis.

**Table 6:** Probiotics and postbiotic regulate cell apoptosis to alleviate infectious diarrhea.

Probiotic-based preparation	Category	Probiotic type	The specific molecular mechanism of apoptosis	References
*Bacillus clausii* Mixed strains (*O/C, T, SIN, N/R*) and superserum	Probiotic Postbiotic	*Bacillus*	Human: Apoptosis may be associated with activation of the cell TLR3 pathway, involving down-regulation of NF-κB1, TRAF6, and MyD88 expression	Paparo et al. (132)
Heat killing and spore forms of *Bacillus subtilis* Gbi-*30*, *Bacillus indiensis ATCC 6633* and *Bacillus coagulans IBRC*-M *10981*	Probiotic	*Bacillus*	Human: By inhibiting the growth of pathogenic bacteria	Kawarizadeh et al. (134)
*LGG* and *MLGG*	Probiotic	*Lactobacillus*	Human: Possibly inhibiting the NF-kB signaling pathway, leading to down-regulation of caspase-3 expression	Buccigrossi et al. (129)
*LGG*	Probiotic	*Lactobacillus*	Animals: The up-regulation of Bcl-2 gene expression and the down-regulation of Bax gene expression	Chakravortty et al. (131)
*L. paracasei ATCC334*, which produces recombinant 3D8 single-chain variable region protein	Postbiotic	*Lactobacillus*	Animals: The expression of anti-apoptotic protein survivin was increased	Hoang et al. (133)

## 8 Probiotic, postbiotic, and safety

Probiotics and postbiotics offer numerous health benefits; however, their potential adverse effects in specific conditions should not be overlooked. First, individuals with underlying diseases and immunocompromised conditions may be at risk of infections when consuming probiotics, particularly those with weakened immune systems, such as chemotherapy patients, pediatric patients, and individuals with HIV (135). In some cases, commercially available probiotics have been associated with infections, such as *Lactobacillus rhamnosus*, which has been reported to cause bacteremia (136). Second, excessive or long-term probiotic consumption may lead to mild gastrointestinal symptoms, such as bloating and indigestion, particularly in individuals with inflammatory bowel disease (IBD). However, these symptoms generally subside as the gut microbiota adapts (137). Furthermore, probiotics may influence drug interactions, potentially reducing the efficacy of concomitant medications. For example, in antibiotic-associated diarrhea, probiotics may delay the restoration of normal gut microbiota (138).

It is also important to consider the potential negative effects of postbiotics. Certain postbiotics may influence autoimmune diseases by either enhancing or suppressing the immune system (139). Additionally, probiotics may be involved in the production of toxic metabolites, such as histamine, which can trigger allergic reactions, headaches, and itching. Recent studies have shown that *Lactobacillus reuteri* plays a role in histamine activation and metabolism, activating the H2 receptor to exert anti-inflammatory effects (140). Moreover, other studies suggest that while the colibactin-producing *Escherichia coli strain Nissle 1917 (EcN1917)* exhibits significantly reduced genotoxic activity compared to other *EcN strains*, it may still increase the likelihood of hazardous mutations through mutagenic mechanisms (141).

In summary, for the general population, probiotics and postbiotics can serve as beneficial dietary supplements for clinical applications. However, their use should be approached with caution in immunocompromised individuals, critically ill patients, infants, and those with severe allergies due to potential risks (142).

## 9 Conclusions and future prospects

Growing evidence indicates a growing interest in probiotic preparations for improving gastrointestinal health. As viable candidates for treating digestive diseases, probiotics exhibit diverse mechanisms and beneficial effects, including modulating immunity, reducing inflammation, improving gut health, alleviating oxidative stress, and regulating apoptosis, with minimal safety risks (5, 143). This review systematically analyzes the ability of different probiotic types and strains to regulate apoptosis, both individually and synergistically. Different strains of the same species, probiotics themselves, and postbiotics may exhibit varying anti-apoptotic effects in disease development and progression. These effects may collectively influence specific signaling pathways and targets, particularly membrane receptors, Bcl-2 family proteins, mitochondria, and caspases. Currently, most research has been conducted in animal models, with only a limited number of studies focusing on apoptosis in cancer at the *in vitro* cellular level. Due to interspecies differences, the complexity of how probiotics regulate apoptosis in human diseases remains incompletely understood. Furthermore, some studies lack comprehensive evidence supporting the regulation of apoptosis. Therefore, careful interpretation of study results is crucial. These phenomena can be partially attributed to the intrinsic properties of probiotics, as well as the high diversity of the human gut microbiota, shaped by factors such as population, sex, diet, and other variables. It is essential to rigorously screen for effective and safe probiotics and postbiotics, either administered through fecal microbiota transplantation (FMT) (144) or directly demonstrating therapeutic effects in animal studies. A systematic transition from small-scale safety assessments to large-scale efficacy validation is necessary to confirm their effectiveness and safety in humans.

Finally, probiotics hold promise as dietary supplements for advancing the treatment of digestive diseases, marking a step toward precision and personalized medicine. Most importantly, further clinical studies are needed to validate the beneficial effects observed in animal models.
